# Unveiling Oxygen K-Edge and Cobalt L-Edge Electron Energy Loss Spectra of Cobalt Hydroxide and Their Evolution under Electron Beam Irradiation

**DOI:** 10.3390/nano13202767

**Published:** 2023-10-16

**Authors:** Jong Hyeok Seo, Ji-Hwan Kwon

**Affiliations:** 1 Korea Research Institute of Standard and Science, Daejeon 34113, Republic of Korea; j.h.seo@kriss.re.kr; 2 Department of Nano Convergence Measurement, Korea University of Science and Technology, Daejeon 34113, Republic of Korea

**Keywords:** transmission electron microscopy, electron energy loss spectroscopy, electron beam damage, cobalt hydroxide

## Abstract

Cobalt hydroxides, Co(OH)_2_, have attracted considerable attention due to their diverse applications in the fields of energy and the environment. However, probing the electronic structure of Co(OH)_2_ is challenging, mainly due to its sensitivity to electron beam irradiation. In this study, we report the unperturbed O K-edge and Co L-edge for Co(OH)_2_ by electron beam damage and investigate the electronic structure transformation of Co(OH)_2_ under electron beam irradiation, using low current electron energy loss spectroscopy. In particular, the O K-edge pre-peak at 530 eV, which is not found in the undamaged Co(OH)_2_, begins to appear with an increasing electron beam current. In addition, the Co L-edge peak shifts to a higher energy, close to Co_3_O_4_, indicating that the localized phase transition within Co(OH)_2_ leads to the formation of Co_3_O_4_.

## 1. Introduction

Transition metal hydroxides have attracted interest for various applications, such as batteries, water splitting, and nanomedicine [1,2,3]. Among transition metal hydroxides, cobalt hydroxide, Co(OH)_2_, has received considerable attention as an electrochemical catalyst for oxygen evolution reaction (OER), because of its electrocatalytic activity and efficient ion intercalation [4,5]. Co(OH)_2_ exists in two phases depending on its structure: α-Co(OH)_2_ and β-Co(OH)_2_ phase. The β-Co(OH)_2_ phase has a brucite-like arrangement, with hydroxyl ions packed hexagonally and Co(II) ions occupying the octahedral site. The α-Co(OH)_2_ phase, on the other hand, resembles a hydrotalcite-like structure, where positively charged Co(OH)_2-*x*_ layers are present. These layers are accompanied by charge-stabilizing anions, such as NO^3−^, CO_3_^2−^, Cl^−^, etc., in the interlayer space [6]. The α-Co(OH)_2_ phase has better electrochemical activity than β-Co(OH)_2_ due to the expended interlayer spacing and intercalated anions [4,5].

Scanning transmission electron microscopy (STEM)–electron energy loss spectroscopy (EELS) measurements have become increasingly popular in atomic structure and chemical analysis as an effective method for characterizing materials in various fields, including for catalysis, batteries, and biotechnology [7,8,9]. However, one critical drawback of this STEM–EELS technique is electron beam-induced damage to the sample [10,11,12,13]. Electron beam-induced damages manifest through a range of effects, including atomic displacement, electron-beam sputtering, radiolysis, heating, and electrostatic charging [14]. These effects hinder the microscope’s ability to fully observe the original structural properties of materials.

Co(OH)_2_ is a material that is highly sensitive to electron beam irradiation. Numerous investigations using transmission electron microscopy have been conducted to explore the structural characteristics of Co(OH)_2;_ however, the effect of electron beam irradiation has not been seriously considered. Only recently has a study demonstrated the impact of electron beam irradiation in inducing structural modifications from Co(OH)_2_ to Co_3_O_4_ [15]. However, there is still a lack of EELS results for Co(OH)_2_, and the reported EELS results vary [16,17], which may be attributed to experimental challenges due to the electron-sensitivity of Co(OH)_2_. It is worth noting that electron beam damage could be more severe in EELS experiments than in imaging because spectroscopy typically requires a longer dwell time than imaging. Therefore, great caution must be taken with electron beam-sensitive materials for EELS experiments.

In this study, we present the undamaged spectra of the O K-edge and Co L-edge for Co(OH)_2_, obtained through low-current EELS. In addition, we demonstrate the evolution of the electronic structure of Co(OH)_2_ under electron beam irradiation in TEM. The low-current EELS experiment (screen current below 30 pA) showed no pre-peak at 530 eV in O K-edge, an observation not reported prior. Previously reported O K-edge spectra for Co(OH)_2_ displayed a pre-peak at 530 eV, which may have been caused by electron beam damage. The electron beam currents exceeding 30 pA induced changes in the O K-edge and the Co L-edge in Co(OH)_2_. The Co L-edge underwent an energy shift to the Co_3_O_4_ position, which was accompanied by the emergence of the O K pre-edge at 530 eV, indicating a phase transition from Co(OH)_2_ to Co_3_O_4_ under electron beam irradiation.

## 2. Experimental Methods

### 2.1. Materials Synthesis

The α-Co(OH)_2_ and β-Co(OH)_2_ particles were prepared under ambient conditions following a previous study [18]. To synthesize α-Co(OH)_2_, a mixture of cobalt chloride hexahydrate (10 mM, CoCl_2_·6H_2_O, Sigma-Aldrich, St. Louis, MO, USA), sodium chloride (50 mM, NaCl, DUKSAN, Ansan, Korea), and hexamethylenetetramine (60 mM, C_6_H_12_N_4_, 99.0%, Sigma-Aldrich, St. Louis, MO, USA) was dissolved in 150 mL of a 1:9 blend of ethanol and deionized water. This solution was then heated in an oil bath at 90 ℃ for one hour, resulting in a suspension containing green particles. The suspended product was subsequently centrifuged at 3500 rpm for 15 min, followed by multiple washes using deionized water and anhydrous ethanol. The final green particles were dried completely at room temperature. The synthesis procedure for β-Co(OH)_2_ mirrored that of α-Co(OH)_2_, with the exception that sodium chloride (NaCl) was omitted from the initial solution. This modification resulted in the formation of pink powder of β-Co(OH)_2_. The Co_3_O_4_ was synthesized through the annealing of α-Co(OH)_2_ at 500 °C for a duration of 3 h under atmospheric conditions.

### 2.2. Electron Microscopy

Scanning transmission electron microscopy (STEM) experiments were conducted using a Thermo Fisher Scientific Spectra 300 operating at an accelerated voltage of 300 kV. EELS experiments were carried out using a Gatan Continuum equipped with a K3 direct electron detector. The electron dose rate for EELS was 4.77 × 10^7^ e^−^/Å^2^∙s. The energy spread of the zero-loss peak (ZLP) was measured at ~1 eV full-width at half-maximum (FWHM). The dispersion was 0.18 eV/ch, and a convergence angle of 22.7 mrad and a collection angle of 64.2 mrad were used. Automatic ZLP alignment was enabled to correct the energy shift during the acquisition time in dual EELS mode. To minimize electron beam damage, EELS experiments were performed in the low-current condition; the screen current was in the range of 1–100 pA and the pixel time for acquisition was 0.1 s. The EELS area scans were performed at 50 × 50 pixels with a step size of 5 nm. The final spectrum was obtained by averaging 2500 spectra. For the sample, the cobalt hydroxides and cobalt oxide powders were effectively dispersed in ethanol and deposited on a lacey carbon grid.

## 3. Results and Discussion

**Figure 1:**
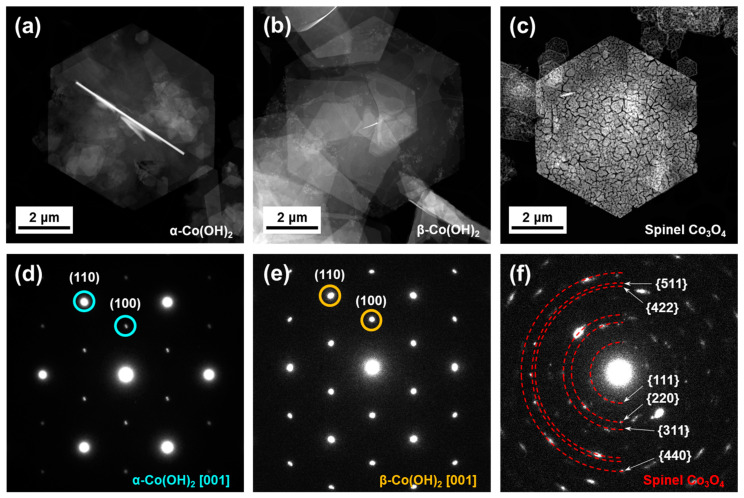
STEM images and typical selected area electron diffraction patterns of representative (**a**,**d**) α-Co(OH)_2_, (**b**,**e**) β-Co(OH)_2_, and (**c**,**f**) spinel Co_3_O_4_ microplates, respectively.

Figure 1 illustrates the scanning transmission electron microscopy (STEM) images along with the selected area electron diffraction (SAED) patterns for α-Co(OH)_2_, β-Co(OH)_2_, and spinel Co_3_O_4_, respectively. In Figure 1a,b, the STEM images show hexagonal microplates of α-Co(OH)_2_ and β-Co(OH)_2_, possessing an average dimension of approximately 6 μm. However, the spinel Co_3_O_4_ hexagonal microplate, synthesized through the annealing of Co(OH)_2_, exhibits granular characteristics accompanied by multiple cracks, as evident in Figure 1c. The SAED patterns reveal that both α-Co(OH)_2_ and β-Co(OH)_2_ consist of a single crystalline phase, while Co_3_O_4_ displays a polycrystalline phase. The combined results from STEM and SAED confirm the successful high-quality synthesis of α-Co(OH)_2_, β-Co(OH)_2_, and spinel Co_3_O_4_, without the presence of any other phases.

**Figure 2:**
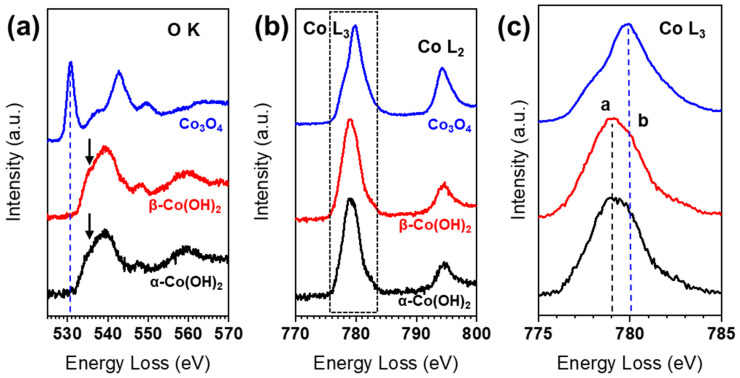
(**a**) EELS O K-edge and (**b**) Co L_3,2_-edge of α-Co(OH)_2_, β-Co(OH)_2_, and Co_3_O_4_, respectively. (**c**) Enlarged spectra for the Co L_3_-edge from the dotted box in (**b**). Each spectrum is shifted vertically for better comparison.

Figure 2 shows the O K-edge and Co L-edge spectra of α-Co(OH)_2_, β-Co(OH)_2_, and Co_3_O_4_, respectively. The spectra of α-Co(OH)_2_ and β-Co(OH)_2_ show distinct features at the O K- and Co L-edges, in contrast to the spectrum of Co_3_O_4_. However, the difference between the α- and β-Co(OH)_2_ spectra was minimal. For Co_3_O_4_, the pre-peak of the O K-edge is located at ~530 eV, while the pre-peak of the O K-edge for Co(OH)_2_ cannot be found, but only a small bump appears at ~534 eV. In the transition metal oxide, the O K-edge pre-peak contributes to the hybridization of O-2p with transition metal 3d states [19]. Previously, several EELS results have been reported for Co(OH)_2_ in which the O K-edge possesses a pre-peak close to 530 eV, as in the case of Co_3_O_4_, indicating the fact that the phase change occurred due to electron beam irradiation [15]. The main peak for the O K-edge is located at ~542 eV in Co_3_O_4_, while the main peak is shifted, towards lower energy, to 539 eV in Co(OH)_2_ marked by the arrows in Figure 2a. The Co L-edge consists of L_3_ and L_2_ white lines, which originate from the transitions of 2p_3/2_ to 3d_3/2_3d_5/2_ and 2p_1/2_ to 3d_3/2_, respectively. These L_3_ and L_2_ white lines are affected by the valence states of the transition metal, due to their relationship to unoccupied states in the transition metal 3d band. Similar to the O K-edge, α-Co(OH)_2_ and β-Co(OH)_2_ show almost identical Co L-edges, but clearly different peak positions and shapes from Co_3_O_4_, as shown in Figure 2b,c. The peak of the Co L_3_-edge of Co(OH)_2_ is at ~779 eV (peak a), and the shoulder peak b is found at ~780 eV. Note that the shoulder peak b in Co(OH)_2_ is almost coincident with the Co L_3_-edge peak for Co_3_O_4_. The white line ratios (L_3_/L_2_) are calculated to be 4.5 for Co(OH)_2_ and 2.6 for Co_3_O_4_, in agreement with previously reported values [20].

**Figure 3:**
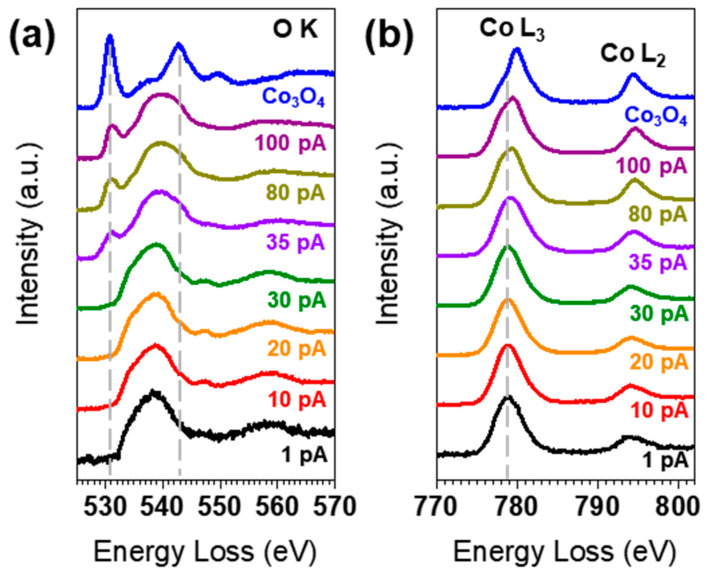
Changes in (**a**) the O K-edge and (**b**) the Co L-edge with increasing electron beam current.

To investigate changes in electronic structure upon electron beam irradiation, we systematically measured the O K- and Co L-edges by varying the beam current. Figure 3 shows the evolution of the O K- and Co L-edges upon electron beam irradiation. At the electron beam current of 1 pA, the pre-peak of the O K-edge is not observed, and this absence persists up to 30 pA. However, at a current exceeding 35 pA, the pre-peak begins to appear at 530 eV. Furthermore, the main peak of the O K-edge at ~538 eV begins to show some spectral weight at 543 eV, which is consistent with the main peak of Co_3_O_4_. The Co L-edge shows a similar trend. The position of the L_3_ peak remains the same up to 30 pA. At currents above 35 pA, the L_3_ peak shifts towards higher energy, indicating a partial conversion of Co(OH)_2_ to Co_3_O_4_ under high-current electron beam irradiation. When the L_3_ peak shifts, the L_3_/L_2_ ratio also changes. The L_3_/L_2_ ratio value decreases from 4.6 at 1 pA to 3.3 at 100 pA, indicating that Co could be oxidized during the EELS experiment.

**Figure 4:**
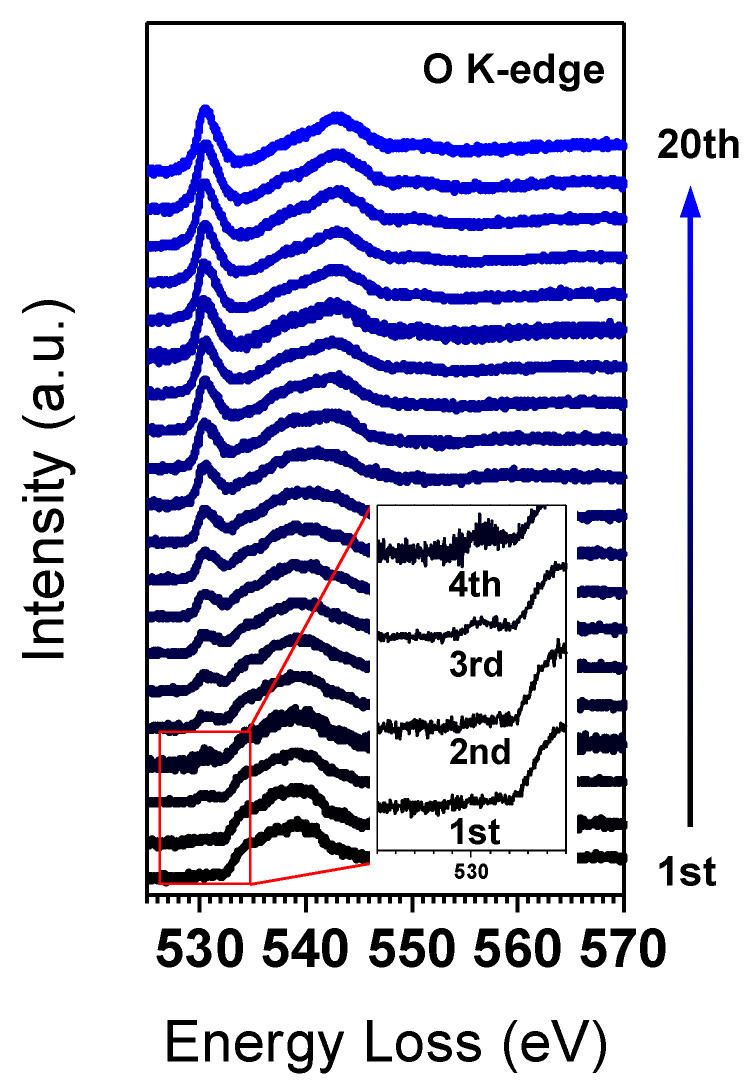
Changes in O K-edge of Co(OH)_2_ over a number of multiple EELS acquisitions.

Finally, we examined the changes in the O K-edge during the phase transition in real time through multiple acquisitions. Figure 4 displays the alterations of the pre-peak in the O K-edge over 20 acquisitions. The scans were conducted within a fixed area of 50 × 50 pixels with a step size of 5 nm. The screen current was 20 pA and each spectrum was scanned for a total of 8 min. In the initial EELS acquisitions, Co(OH)_2_ did not exhibit a pre-peak in the O K-edge, as previously described. However, after the third acquisition, the O K-edge pre-peak, indicative of spinel Co_3_O_4_, began to appear at 530 eV, and its intensity increased with the number of acquisitions. This change suggests that the low-current electron beam also causes phase transition under persistent irradiation.

The phase transition of Co(OH)_2_ to Co_3_O_4_ is observed in the thermal decomposition process, following two steps [21]: (1) Co(OH)_2_ + 0.25O_2_ → 2CoOOH + 0.5H_2_O; (2) 3CoOOH → Co_3_O_4_ + 1.5H_2_O + 0.25O_2_. However, the phase transition induced by electron beam irradiation could follow a different reaction path, due to the different environment in the TEM chamber. The electron beam could easily kick off the hydrogen atoms in Co(OH)_2_, resulting in the formation of CoOOH phases, which had been found in an in situ electron diffraction experiment under electron beam irradiation [15]. However, in this study, the existence of CoOOH was not clearly shown in the O K-edge and Co L-edge EELS results, which could be attributed to the relatively fast phase transition from Co(OH)_2_ to CoOOH, compared to the subsequent phase transition to Co_3_O_4_. It is worth noting that the multiple EELS acquisitions in Figure 4 show continuous change of the O K-edge and the Co L-edge over the irradiation time, and no discernible change that could indicate a CoOOH phase is found during these multiple acquisitions. The CoOOH to Co_3_O_4_ phase transition is anticipated to follow a reaction similar to the thermal decomposition process, and the water and oxygen molecules could be extracted in the gas phases in TEM.

## 4. Conclusions

In this study, we reported the original electronic structure of Co(OH)_2_, and systematically investigated the changes in the electronic structure of Co(OH)_2_ under an electron beam by changing the electron beam current for the EELS experiment. Employing low-current EELS conditions (with an electron current below 30 pA) revealed the unperturbed O-K edge and Co-L edge spectra for Co(OH)_2_, thereby contrasting with previous EELS data. However, the electron beam currents exceeding 30 pA induced alterations in the O-K edge and Co-L edge for both α-Co(OH)_2_ and β-Co(OH)_2_. The O-K pre-edge at 530 eV, indicative of Co_3_O_4_, began to appear with an increasing electron beam current, accompanying the energy shift of the Co-L edge to the position for Co_3_O_4_. These observed EELS spectral changes indicate the facile transformation of Co(OH)_2_ into Co_3_O_4_ under electron beam irradiation. Our results emphasize the need for a cautious interpretation of results from EELS experiments and highlight the potential impact of electron beam influence on the electronic structure of materials.

## Data Availability

The datasets used and/or analyzed during the current study are available from the corresponding author on reasonable request.
